# Trajectories of neighborhood environmental factors and their associations with asthma symptom trajectories among children in Australia: evidence from a national birth cohort study

**DOI:** 10.1007/s40201-022-00824-z

**Published:** 2022-09-13

**Authors:** K M Shahunja, Peter D Sly, M Mamun Huda, Abdullah Mamun

**Affiliations:** 1 UQ Poche Centre for Indigenous Health, Faculty of Medicine, The University of Queensland, Brisbane, Australia; 2 ARC Centre of Excellence for Children and Families over the Life Course, The University of Queensland, Brisbane, Australia; 3 The Queensland Alliance for Environmental Health Sciences (QAEHS), The University of Queensland, Brisbane, Australia; 4 Child Health Research Centre, Faculty of Medicine, The University of Queensland, Brisbane, Australia

**Keywords:** Neighborhood environment, Asthma, Trajectory, Children, Australia

## Abstract

**Purpose:**

This study aims to investigate the prospective associations of neighborhood environmental exposure trajectories with asthma symptom trajectories during childhood developmental stages.

**Methods:**

We considered asthma symptom, neighborhood environmental factors, and socio-demographic data from the “Longitudinal Study of Australian Children (LSAC)”. Group-based trajectory modeling was applied to identify the trajectories of asthma symptom, neighborhood traffic conditions, and neighborhood livability scales (considered for safety and facilities). We used multivariable logistic regression models to assess associations between various neighborhood environmental factors and asthma symptom trajectories.

**Results:**

We included 4,174 children from the LSAC cohort in our study. Three distinct trajectories for asthma symptom were the outcome variables of this study. Among the neighborhood environmental factors, we identified two distinct trajectories for the prevalence of heavy traffic on street, and two trajectories of neighborhood liveability scale. Compared to the ‘Low/no’ asthma symptoms trajectory group, children exposed to a ‘persistently high’ prevalence of heavy traffic on street was also significantly associated with both ‘transient high’ [relative risk ratio (RRR):1.40, 95% CI:1.25,1.58) and ‘persistent high’ (RRR: 1.33, 95% CI:1.17,1.50)] asthma symptom trajectory groups. Trajectory of moderate and static neighborhood liveability score was at increased risk of being classified as ‘transient high’ (RRR:1.16, 95% CI:1.07,1.25) and ‘persistent high’ (RRR:1.38, 95% CI:1.27,1.50) trajectories of asthma symptom.

**Conclusion:**

Exposure to heavy traffic and poor neighborhood liveability increased the risk of having an unfavourable asthma symptom trajectory in childhood. Reducing neighborhood traffic load and improving neighborhood safety and amenities may facilitate a favorable asthma symptom trajectory among these children.

**Supplementary Information:**

The online version contains supplementary material available at 10.1007/s40201-022-00824-z.

## Introduction

Australia is one of the high prevalent countries for childhood asthma globally [1]. Every year, the country bears a substantial healthcare cost to treat the morbidities related to this illness [2]. Asthma is a heterogeneous disease and has several disease prototypes [3]. Although the symptoms cannot be cured permanently, they can be fairly controlled by taking appropriate preventive and therapeutic measures. Thus, due to a lack of appropriate preventive measures and exposure to triggering factors [4, 5], the symptom prevalence may differ and follow distinct trajectories over time [4, 6]. A prior study in Australia showed asthma symptom (as wheezing) in childhood (0–15 years) follows three distinct trajectories as ‘Low/no (69%)’, ‘Transient high (17%)’, and ‘Persistent high a (14%)’ asthma symptom groups [7].

Several environmental factors may trigger and make it difficult to prevent asthma symptoms [8]. Although in-house environmental exposures (such as passive smoking, mold, house dust mites, pets, gas heaters, etc.) [9] could be more common and easily understandable factors responsible for asthma symptoms, some neighborhood environmental factors can also contribute to substantial risk for exacerbating asthma symptoms [8, 10–14]. This neighborhood environment is crucial as it can help adopt and maintain both the physical and mental well-being of a person [15]. For asthma symptoms, some components of the neighborhood environment (such as traffic-related air pollution) [11] can affect the lungs and contribute directly to the disease pathway [16]. A systematic review and meta-analysis by Jingchun et al. reported the risk of emergency department visits for asthma due to the short-term effect of air pollutants (e.g., particulate matter_2.5_) [16]. Socially, an unsafe neighborhood would also be a risk for asthma symptoms exacerbation. A study reported associations among neighborhoods being unsafe and higher asthma prevalence, and poorer asthma control [12]. Other factors, such as low walkability [13] and a lack of sufficient green space [17], may also worsen this condition in other indirect pathways either by facilitating stress [18, 19] or failing to buffer other pollutants [17]. Residential instability, neighborhood deprivation and lack of proper facilities for the inhabitants can also contribute to exacerbating asthma symptoms [20, 21].

Although most of the studies assessed the association between asthma symptoms and several individual components of neighborhood environment [8, 10–14, 17, 20, 21], a composite scale, such as an overall liveability of a neighborhood, would also be associated with asthma symptoms. Liveability is a human perspective regarding the environmental characteristics of a residential area [22]. It reflects the well-being of a community and comprises the many characteristics that make a location desirable [23]. However, there is a scarcity of data regarding the association between neighborhood liveability and asthma symptoms. There is no consensus concerning the essential conditions of a liveable community [24]. However, several scales have been introduced considering several individual facts in neighborhoods to assess how habitable and pleasant a neighborhood is to live in [22, 24, 25]. The Longitudinal Study of Australian Children (LSAC) [26] considered neighborhood liveability scale based on the perception of inhabitants on neighborhood safety, local parks/playground quality, roads/footpaths and street lighting. The study also assessed neighborhood facilities on a scale considering local access to public transport, shops, and other services [26].

Most of these neighborhood environmental factors are dynamic and can change their extent of exposure over time. Thus, variable exposures can influence the trajectories of asthma symptoms over time. It is important to assess the trajectories of these dynamic environmental factors and their impact on the trajectories of asthma symptoms. However, most prior studies have evaluated the immediate effect of the environmental factors on asthma symptoms [12, 16, 18, 19] rather than effects on long-term trajectories of asthma symptoms, especially in childhood. A Canadian study [5] reported an increase in nitrogen dioxide exposure during the antenatal period increased the risk of having a bad asthma symptom trajectory in childhood. However, in that study exposure was measured in the antenatal period only. It is important to know whether continuous exposure during childhood or early life exposure is more indicative of a bad asthma symptom trajectory for all of childhood. Because, although lung development starts in utero, it continues through adolescence and early adulthood [27, 28]. Therefore, environmental insult at any point of this long developmental period of the lung can lead to respiratory morbidity or reduced lung function [27, 29].

There is a scarcity of studies globally that assess the trajectories of these dynamic and modifiable environmental risk factors and their association with asthma symptoms trajectories throughout childhood. We aimed to determine the trajectory of different neighborhood environmental factors and their associations with the trajectories of asthma symptom among children in Australia using the data of a nationally representative cohort [30].

## Methods

### Data source

In this study, we used the data of the Longitudinal Study of Australian Children (LSAC). This is a cross-sequential dual cohort study. The LSAC study is conducted in partnership with the Australian Government Department of Social Services, the Australian Institute of Family Studies (AIFS), and the Australian Bureau of Statistics (ABS) [26]. The study aims to build understandings of child development, inform social policy debate and identify opportunities for intervention and prevention strategies in policy areas concerning children and their families. It is a good source of several neighborhood environmental indicators and different health outcomes, such as asthma symptoms over eight time points (0–15 years of age) and consists of a birth (children born March 2003 to February 2004) and a kindergarten cohort (children born March 1999 to February 2000). The study recruited the participants by sampling through the government medical insurance (Medicare) enrolment database [26]. LSAC collects data biennially, and so far, they have eight waves of data. In our study, we used the data from waves 1–8 of the ‘B’ (birth cohort) cohort of the LSAC study. In this cohort, children were recruited between the ages of 0 and 1 year of age in 2004 (wave 1). Although the LSAC ‘B’ cohort started with 5107 children, the sample size was reduced in every consecutive wave due to attrition. At wave eight, 3127 children remained [26]. The sampling design and field methods of the LSAC study have been described elsewhere [30].

### Analytical sample

Out of 5,107 children, we included 4,174 children from 0 to 15 years of age of either sex as our analytical sample (See result section). We could not include 933 participants from the initial cohort of the LSAC study due to attrition in the following waves or the unavailability of desired information.

### Outcome measurements

We considered three distinct trajectory groups of asthma symptom as ‘low/no (69%)’, ‘transient high (17%)’, and ‘persistent high (14%)’ as our outcome variables for this study. These trajectory groups have been identified from the same participants of this study (LSAC cohort) and described in an earlier study [7].

### Exposures measurements

Several neighborhood environmental factors were the exposure variables in this study. We considered these neighborhood environmental exposures based on literature reviews [8, 10–14, 17, 20, 21] and availability in the LSAC data. We evaluated the neighborhood’s environmental status by assessing its liveability scale, facility scale, traffic conditions on streets, and general conditions of nearby buildings. We also assessed the trajectory groups for ‘prevalence of heavy traffic on street’ and ‘neighborhood liveability score’ and considered their trajectory groups as our exposure variables in the final analysis model. In addition to the LSAC data, we collected neighborhood greenspace [as Normalized Difference Vegetation Index (NDVI)] data from the Bureau of Meteorology, Australia (http://www.bom.gov.au/climate/austmaps/about-ndvi-maps.shtml). We linked these data with the LSAC data and considered it as one of our exposure variables. The methodological approach for collecting and calculating NDVI values is mentioned in the supplemental document (Text ST1). A detailed variable list with a description of the measurements of exposure variables is included in table S1. We considered these environmental variables in three analytical groups according to their exposure time, such as exposure in the first year of life (analytical model 1), the first five years of life (analytical model 2), and finally, the whole follow-up period (birth to 15 years) (analytical model 3). We included exposure in the first year and the first five years of life as these are the critical time points for the rapid structural and functional development of the lungs [31]. Adverse environmental exposure during this period can lead to chronic respiratory morbidity in children and adulthood [29]. There are also no data on the effects of sustained exposure to these environmental factors over the first 15 years of life. Thus, we chose to examine the effects of neighborhood environmental exposures during these three times of life on trajectories of asthma symptom throughout childhood.

### Adjusting variables

The literature suggests that the prevalence of asthma symptoms may vary by sex [32], and ethnicity [32], poor health status or having a comorbid condition [33], pre-term birth, and low birth weight [34]. Additionally, maternal cigarette smoking [35] and maternal asthma during pregnancy [36] contribute to the individual risk of their offspring developing asthma symptoms in childhood. These factors may alter the magnitude of primary exposures (neighborhood environmental factors) on the outcome (asthma symptom trajectories). In our study, children’s factors, including sex, indigenous status, health status, pre-term birth, and current medication for asthma; maternal factors, such as maternal smoking and maternal asthma medication during pregnancy; and family factors such as socioeconomic status according to ‘Socio-Economic Indexes for Areas (SEIFA) economic resources’[37] were adjusted in the multivariable models to determine the independent effects of neighborhood environmental factors on the trajectories of asthma symptoms. A detailed variable list with a description of the adjusting variables is included in table S2.

### Statistical analysis

We have described the baseline demographic characteristics of the study children, the prevalence of asthma symptom, and different neighborhood environmental factors over different time points. The significance between groups was measured by chi-square test (x^2^) for categorical variables and student t-test for continuous variables. We considered the *p-*value < 0.05 as significant in all univariate analyses. However, we used Dunn-Šidàk (38) corrections in multivariable models to control the familywise error rate to be 0.05. Depending on the number of variables in different multivariable regression models, we considered the individual *p*-values (for adjusted model 1: *p* < 0.004, model 2 *p* < 0.003, and 3: *p* < 0.005) as statistically significant. All statistical analyses were undertaken using Stata IC 16.0 (Stata Statistical Software, College Station, Tx, USA) [39].

#### Group-based trajectory modeling

We have conducted group-based trajectory modeling (GBTM) [40, 41] to identify the trajectory groups of two time-variant environmental factors such as ‘heavy traffic on street’ and ‘neighborhood liveability scale’. We could not use GBTM for other neighborhood environmental factors (e.g., neighborhood facilities scale, general condition of nearby buildings, and NDVI) as their prevalence did not change remarkably over time (see result section), and a bivariate analysis found an insignificant association with outcomes in most time points (see result section). A brief description of the statistical procedures for GBTM is mentioned in the supplementary document (Text ST2), and model selection criteria are mentioned in tables S3-4.

#### Model to assess the association between different neighborhood environmental factors and asthma symptom trajectories

We used multinomial logistic regression to assess the associations of different neighborhood environmental factors on the group trajectories of asthma symptom. The reference category for the outcome variable was the ‘low / no’ asthma symptom trajectory group. The odds ratio (OR) and relative risk ratio (RRR) with their 95% confidence intervals were reported accordingly for the bivariable and multivariable results. At first, we did bivariate analyses to assess the unadjusted association between different neighborhood environmental factors and asthma symptom trajectories over different time points (see result section). Later, we constructed three different multivariable models after adjusting potential confounders (see result section). These models were used to see the association between asthma symptom trajectories and different neighborhood environmental factors exposed at 0/1 year (Model 1), over the first 4/5 years (Model 2), and over the first 14/15 years (Model 3). We developed these three models according to the time of exposure. The first and second models (Model 1, 2) attempted to capture the environmental factors exposed during the first year and first 4/5 years of life in a cross-sectional manner. In our final model, we captured the variation of the environmental exposure over childhood (0–15 years) as their trajectories and assessed their associations with asthma symptom trajectories.

### Dealing with missing values

We used multiple imputations to handle the missing values under the assumption of missing at random [42]. Imputations were done for the missing independent and adjusting variables using chained equations. A sensitivity analysis was conducted by comparing the output of the multivariable regression between complete case and imputed data for all three models (Tables S5–7). There were also attritions of in LSAC cohort in different waves, and a deficit of sufficient information for the outcome variables of our study. Thus, our analytical sample is less and different from the LSAC original sample which may create selection bias and affects the association estimates. To control this selection bias [43], we applied the inverse probability weighting (IPW) technique [44] in our regression models and showed it in a sensitivity analysis (supplementary tables S8-10) before and after incorporating the IPW technique. However, the estimates did not change much after incorporating IPW technique. Thus, we have shown the results of multivariable models without IPW technique in the result section of this paper.

## Results

**Fig. 1:**
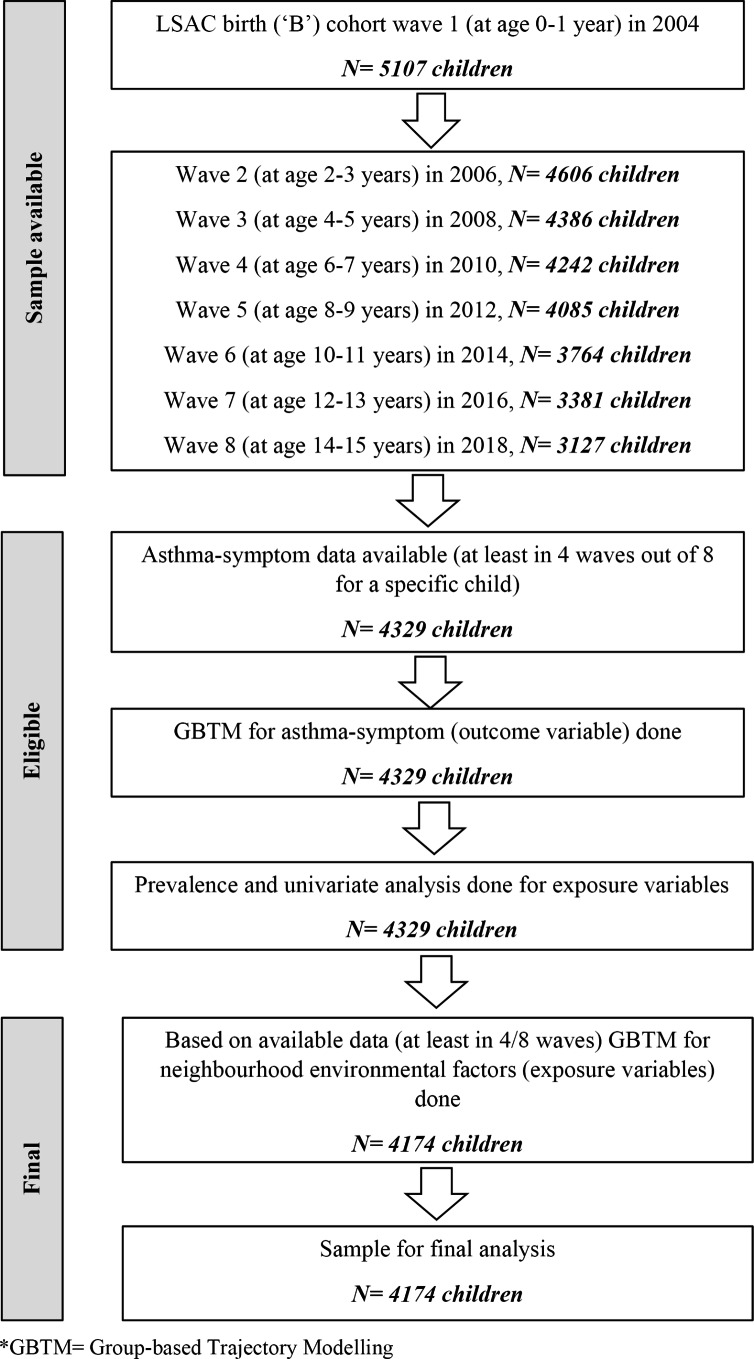
Study flow chart for sample selection

We included 4,174 children out of the 5,107 from the LSAC ‘B’ cohort for this study. Figure 1 illustrates how the analytical sample (N = 4,174) was obtained from the initial cohort of the LSAC study. A total of 4,329 children met the eligibility for GBTM to assess the asthma symptom trajectory groups. The GBTM process and the characteristics of the asthma symptom trajectory groups were described in an earlier study [7].

We compared the baseline characteristics across the three different trajectory groups of asthma symptom in our study. We have described the participants’ socio-demographic and health status, including sex, ethnicity, birth weight, gestational age, presence of any medical condition, maternal smoking during pregnancy, socioeconomic conditions, etc., in Table 1. Most of these characteristics were significantly different across the three trajectory groups of asthma symptom.

We also compared the baseline characteristics between the analytic sample (n = 4,174) and the rest of the participants (n = 933) of the initial cohort of the LSAC study (Table S11). Among the 4,174 children (analytic sample) in our study, 51% were male, and 3% came from Indigenous backgrounds. The proportion of Indigenous people, the presence of any medical condition, and maternal smoking during pregnancy were significantly higher in non-included participants (Table S11).

**Table 1:** Baseline characteristics of the analytical cohort

Variables	Total *N = 4174*	Group 1 *n = 3185* (76)	Group 2 *n = 454* (11)	Group 3 *n = 535* (12)	*P* value
Male sex (n, %)	2145(51)	1591(50)	259(57)	295(55)	0.003
Ethnicity- Indigenous (n, %)	133(3)	85(3)	22(5)	26(5)	0.003
Low birth weight (n, %)	214/4152 (5)	153/3172 (5)	30/449 (7)	31/531 (6)	0.186
Pre-term birth (n, %)	262/4139 (6)	178/3156 (6)	51/451 (11)	33/532 (6)	< 0.001
Presence of any medical condition_ yes (n, %)	216(5)	110(3)	45(10)	61(11)	< 0.001
Maternal smoking during pregnancy_ yes (n, %)	546/3663 (15)	355/2821 (13)	92/389 (24)	99/453 (22)	< 0.001
Maternal asthma medication during pregnancy_ yes (n, %)	150/4165 (4)	90/3178 (3)	23/452 (5)	37(7)	< 0.001
SEIFA economic resources					
Lowest quantile	1519(36)	1161(36)	159(35)	199(37)	0.023
Second quantile	847(20)	636(20)	88(19)	123(23)	
Third quantile	875(21)	654(20)	121(27)	100(19)	
Highest quantile	933(22)	734(23)	86(19)	113(21)	

Comparisons done between group 1 vs. group 2; and group 1 vs. group 3); Group 1 = Low/no asthma symptom trajectory group; Group 2 = Transient high asthma symptom trajectory group; group 3 = Persistent high asthma symptom trajectory group; SE = standard error; Low birth weight = Birth weight <2500gm; Pre-term = Gestational age <37 weeks; SEIFA = Socio-Economic Indexes for Areas

In this study, we assessed the prevalence of asthma symptom and several neighborhood environmental factors at different time points (ages of the participants). The overall prevalence of asthma symptom varies from 9–25% at different time points. The prevalence was higher in the first 6/7 years of age, with a peak of 25% at ages 2/3. Males had a higher prevalence of asthma symptom than females at almost all time points. Our cohort showed that none of the neighborhood environmental factors showed any remarkable changes in their prevalence over the 15 years or any specific trend (e.g., gradual increase or decline). Instead, they showed fluctuations in their prevalence during this time frame (Table S12)

Bivariate analyses showed that the ‘heavy traffic on street’ was significantly associated with the ‘transient high’ and ‘persistent high’ asthma symptom trajectory group compared to the ‘low/no’ asthma symptom trajectory group (control) at most of the time points (Table S13). The condition of the neighborhood liveability scale also showed the same association with the asthma symptom trajectory groups. However, other factors such as ‘neighborhood facility scale’, ‘general condition of nearby buildings’, and ‘mean NVDI values’ of that area did not show a statistically significant association with the asthma symptom trajectories across the time-points (Table S13)

**Fig. 2:**
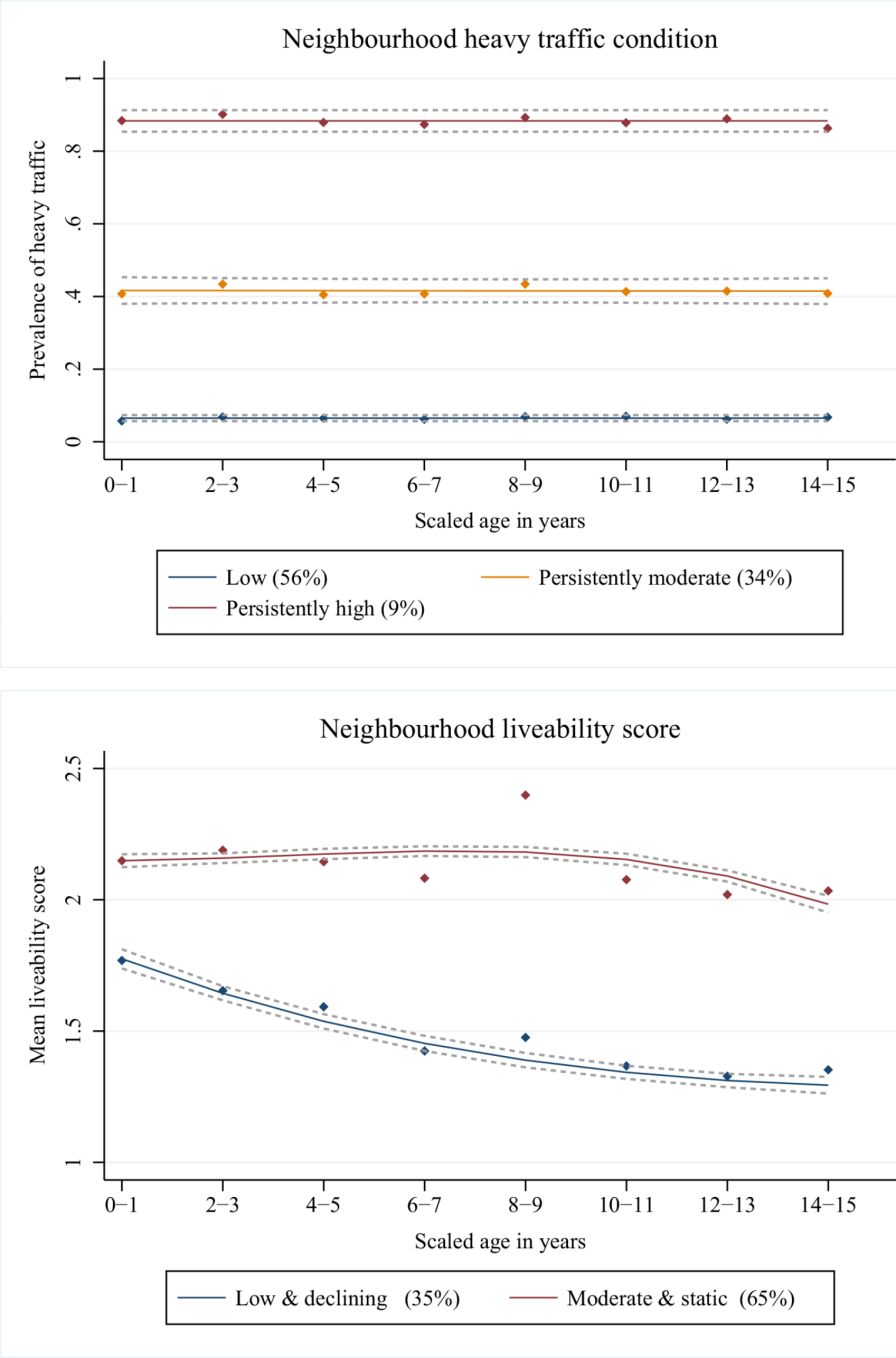
Trajectory groups for neighborhood environmental factors

We used GBTM for neighborhood status of ‘heavy traffic on street’ and ‘liveability scale’. After the same model-fitting approach as GBTM for asthma symptom trajectories, ‘heavy traffic on street’ showed three groups’ trajectories, and neighborhood ‘liveability score’ showed two-groups trajectories in their best-fitting models (Tables S3-4). The prevalence of heavy traffic on street nearby the participants’ residents showed ‘low (prevalence < 10%)’, ‘persistently moderate (prevalence of about 40%)’ and ‘persistently high (prevalence > 80%) trajectory groups. Most of the children belonged to the low prevalence group (56%), 34% to moderate, and only 9% to the high prevalence group from ‘heavy traffic on street’ (Fig. 2). About one-third of the children in the study belonged to low and declining mean liveability scores, while the other two-thirds had a moderate mean liveability score (a lower number on the scale indicates better neighborhood condition) that was almost static over time (Fig. 2). We also found that 22% of children had both ‘persistently moderate heavy traffic trajectory’ and ‘moderate & static neighborhood liveability trajectory’. Another 7% of children had ‘persistently high heavy traffic trajectory’ and ‘moderate & static neighborhood liveability trajectory’ in common (table S14)

After adjusting the potential covariates, we constructed three different multivariable models (based on exposure time) to assess the independent associations between asthma symptom trajectory groups and different neighborhood environmental factors. A relative risk ratio was calculated using multinomial logistic regression adjusted for participants’ sex, Indigenous status, any medical condition, current asthma medications, pre-term birth, asthma medications of mother during pregnancy, cigarette smoking of mother during pregnancy, and SEIFA economic resources index for parents for all multivariable models

Model 1 shows the association of asthma symptom trajectory groups with exposure to various neighborhood environmental factors in the first year of life. Among the five environmental factors, ‘heavy traffic on street’ and ‘neighborhood liveability scale’ showed a statistically significant association with ‘persistent high’ asthma symptoms trajectory group in the unadjusted model. However, in the adjusted analysis, these were not associated significantly (Table 2).

**Table 2:** Association of different neighborhood environmental factors (exposed in age 0/1 year) with asthma symptom trajectories (Multivariable model 1)

Variables	Unadjusted (N = 4174)		Adjusted* (N = 4174)	
	Transient high	Persistent high	Transient high	Persistent high
Heavy traffic on street				
Disagree	1	1	1	1
Agree	1.16(0.92,1.46)	**1.36(1.11,1.67)**	1.13(0.90,1.43)	1.31(1.07,1.62)
General condition of nearby buildings				
Good	1	1	1	1
Bad	1.03(0.46,2.31)	0.56(0.22,1.44)	0.81(0.34,1.87)	0.41(0.15,1.09)
Neighborhood liveability scale	1.24(1.00,1.53)	**1.47(1.20,1.80)**	1.15(0.92,1.43)	1.33(1.08,1.63)
Neighborhood facilities scale	1.02(0.89,1.18)	1.03(0.90,1.17)	1.01(0.87,1.16)	1.01(0.88,1.16)
NDVI value of the area	1.22(0.51,2.93)	0.56(0.24,1.26)	1.17(0.47,2.89)	0.54(0.23,1.26)

Association presented as relative risk ratio and their 95% confidence interval, 1 = Reference value;

Reference group for trajectory is ‘No/low asthma symptom trajectory group’; Bold numbers represent statistically significant values after ‘Dunn-Šidàk test’ for p-values (p < 0.010 was considered for the unadjusted model and p < 0.004 for the adjusted model as statistically significant);

*Adjusted for sex, Indigenous status, SEIFA economic resources, any medical condition, pre-term birth, asthma medication of mother during pregnancy, maternal smoking during pregnancy

Table 3 shows the association of asthma symptom trajectory groups with different environmental factors exposed over the first 4/5 years. The adjusted result showed that a positive response to ‘heavy traffic on street’ recorded more than one time point in the first 4/5 years of life incurred significantly higher risk to be associated with ‘transient high’ and ‘persistent high’ asthma symptom groups when compared with children never exposed to ‘heavy traffic on street’. Children who had comparatively higher mean liveability score during the first 4/5 years have a higher risk of belonging ‘persistent high’ asthma symptom trajectory group, and higher mean facilities scale associated with ‘transient high’ asthma symptom trajectory group compared to ‘low/no’ asthma symptom trajectory group (Table 3).

**Table 3:** Association of different neighborhood environmental factors (exposed over age 4/5 years) with asthma symptom trajectories (Multivariable model 2)

Variables		Unadjusted (N = 4174)		Adjusted* (N = 4174)	
	Group %	Transient high	Persistent high	Transient high	Persistent high
Heavy traffic on street_ agree					
Never	55%	1	1	1	1
1–2 time points	37%	**1.31(1.21,1.41)**	**1.26(1.17,1.35)**	**1.28(1.19,1.38)**	**1.24(1.15,1.34)**
All 3 time points	8%	**1.33(1.17,1.52)**	**1.45(1.29,1.63)**	**1.35(1.18,1.54)**	**1.47(1.29,1.68)**
General condition of nearby buildings_ bad					
Never	97%	1	1	1	1
1–2 time points	3%	1.15(0.95,1.40)	1.02(0.84,1.22)	0.93(0.76,1.14)	0.85(0.69,1.04)
All 3 time points	< 1%	Not done^#^	Not done^#^	Not done^#^	Not done^#^
Neighborhood liveability scale (mean of 3 time points)		1.05(0.95,1.15)	**1.50(1.38,1.64)**	0.97(0.88,1.07)	**1.37(1.24,1.51)**
Neighborhood facilities scale (mean of 3 time points)		**1.16(1.08,1.23)**	1.01(0.95,1.07)	**1.16(1.08,1.23**)	1.05(0.98,1.12)
NDVI value of the area (mean of 3 time points)		1.16(0.82,1.65)	0.68(0.49,0.94)	1.12(0.78,1.60)	0.81(0.56,1.17)

Association presented as relative risk ratio and their 95% confidence interval, 1 = Reference value;

Reference group for trajectory is ‘No/low asthma symptom trajectory group’; Bold numbers represent statistically significant values after ‘Dunn-Šidàk test’ for p-values (p < 0.010 was considered for the unadjusted model and p < 0.003 for the adjusted model as statistically significant);

^#^Analysis not done due to very low cell frequency

*Adjusted for sex, Indigenous status, SEIFA economic resources, any medical condition, pre-term birth, asthma medication of mother during pregnancy, maternal smoking during pregnancy, current asthma medication

Our final model (Model 3) shows the association of asthma symptom trajectory groups with exposure to different neighborhood environmental factors over the first 14/15 years of life for the studied children. We assessed the associations between asthma symptom trajectory groups and trajectory groups of two different environmental factors. In both the unadjusted and adjusted models, trajectory groups with a higher prevalence of heavy traffic on street were associated with both ‘transient high’ and ‘persistent high’ trajectory groups of asthma symptom. Compared to the low prevalence group, children with a persistently moderate prevalence of ‘heavy traffic on street’ had 21% more risk associated with ‘transient high’, and 29% more risk associated with ‘persistent high’ asthma symptom group. The group with the persistently high prevalence of heavy traffic on the street had more risk (40%) of belonging to the ‘transient high’ asthma symptom group (Table 4). Children with a ‘moderate and static’ mean livability score also had significantly higher risk associated with both ‘transient high’ and ‘persistently high’ asthma symptom trajectory groups (Table 4)

**Table 4:** Association of different neighborhood environmental factors (exposed over age 14/15 years) with asthma symptom trajectories (Multivariable model 3)

Variables	Unadjusted (N = 4174)		Adjusted* (N = 4174)	
	Transient high	Persistent high	Transient high	Persistent high
Prevalence of heavy traffic on street (trajectory groups)				
Low	1	1	1	1
Persistently moderate	**1.29(1.19,1.39)**	**1.37(1.27,1.47)**	**1.21(1.12,1.31)**	**1.29(1.20,1.40)**
Persistently high	**1.43(1.28,1.61)**	**1.36(1.22,1.51)**	**1.40(1.25,1.58)**	**1.33(1.17,1.50)**
Neighborhood liveability score (trajectory groups)				
Low & declining	1	1	1	1
Moderate & static	**1.22(1.14,1.32)**	**1.42(1.32,1.52)**	**1.16(1.07,1.25)**	**1.38(1.27,1.50)**

Association presented as relative risk ratio and their 95% confidence interval, 1 = Reference value;

Reference group for trajectory is ‘No/low asthma symptom trajectory group’; Bold numbers represent statistically significant values after ‘Dunn-Šidàk test’ for p-values (p < 0.025 was considered for the unadjusted model and p < 0.005 for the adjusted model as statistically significant);

*Adjusted for sex, Indigenous status, SEIFA economic resources, any medical condition, pre-term birth, asthma medication of mother during pregnancy, maternal smoking during pregnancy, current asthma medication

## Discussion

Our study identified the Trajectories of two neighborhood environmental factors that showed distinct patterns throughout childhood. Relatively bad trajectories for the condition of ‘heavy traffic on street’ and ‘neighborhood liveability score’ were significantly associated with both transient and persistently high prevalence of asthma symptom trajectory groups. Children had a relatively higher chance of belonging to the persistently high prevalence of asthma symptom trajectory group when they were exposed to these two detrimental neighborhood environmental factors in their early years of life.

In this study, we tried to assess the association of five important neighborhood environmental factors with asthma symptom trajectories. These neighborhood environmental factors showed positive associations with asthma symptom in prior studies [10–14], but none assessed their changing patterns and impacts on asthma symptom trajectories. Considering the prevalence of these factors, or average values over different time points, none showed any remarkable change, or followed any distinct trend during the eight time points over the first 15 years of our participants’ ages. Although we did not find any data on nationwide prevalence and trend of a composite scale of neighborhood environment (such as liveability scale, facility scale), data on some individual environmental indicators were reported earlier. According to the Australian Government Department of the Environment and Energy report, the percentages of total per kilometer urban travel by private vehicles and public buses were almost static over the last few decades [45]. Other than the LSAC study, the General Social Survey (GSS) by the Australian Bureau of Statistics also reported that perceived neighborhood safety did not change much in 2006, 2010, and 2014 [46]. So, these can explain the generalizability of the LSAC data, especially regarding the prevalence and trends of these environmental factors.

The presence of heavy traffic on neighborhood streets is a proxy indicator for assessing neighborhood traffic-related air pollution [47]. Motor vehicles emit large quantities of pollutant gases and particles such as carbon dioxide (CO_2_ ), carbon monoxide (CO), hydrocarbons (HC), nitrogen oxides (NO_2_), ozone, and particulate matter (PM) etc., which are harmful [48]. These are the main pollutants from traffic-related air pollution (TRAP) [48], and the amount of these pollutant gases would be directly proportional to the number of traffic on the street. Decades of research have shown that air pollution can exacerbate asthma symptoms [48–50]. These pollutants (e.g., ozone, nitrogen dioxide, ozone, nitrogen dioxide, and PM_2.5_) can cause airway inflammation and airway hyper-responsiveness, which eventually trigger asthma symptoms [51, 52]. Pollutant exposure can also initiate oxidative stress, which is a feature of severe asthma [52]. Therefore, exposure to these pollutants is expectedly correlated with exacerbations and possibly even the onset of asthma symptoms [52]. Although several prior studies reported the association between TRAP and the onset or exacerbations of asthma symptoms, it is important to know the long-term trajectories of TRAP (as a condition of heavy traffic on street) and asthma symptoms in a defined population, which we attempted to investigate in this study. While our study is the first in Australia to assess this association, a Canadian study previously showed that exposure to a higher amount of nitrogen dioxide (one of the pollutant gases from TRAP) is significantly associated with chronic asthma trajectories [5], which is a similar finding to our study.

The LSAC study considered neighborhood safety and some neighborhood amenities (such as good parks, playgrounds and play spaces, footpaths, roads, and street lighting) to measure the composite scale of neighborhood liveability. Although the LSAC study did not consider neighborhood traffic condition as an indicator for neighborhood liveability scale, in our study, we found a considerable number of children belonged to both bad trajectories of neighborhood liveability and neighborhood traffic conditions in common. Several Australian cities have been named ‘the world’s most liveable city’, and we also found that the overall liveability score is lower (a higher score indicates bad liveability status) in Australia. However, we found a significant association between the persistently moderate or high number of liveability scores and persistent and high asthma symptom trajectories among children in Australia. Although it is not well understood how poor neighborhood liveability is associated with bad trajectories of asthma symptom, individual components of the liveability scale might contribute to this. For example, some studies have reported an association between neighborhood safety and asthma symptoms. Additionally, being unsafe was associated with higher asthma prevalence or poorer asthma control in children [12, 18]. Other neighborhood facilities, such as low walkability was found as a risk for asthma symptoms in children [13]. An unsafe neighborhood or a lack of expected neighborhood amenities can cause mental stress for children as well as for their parents [18] which may contribute to higher asthma rates and worsen asthma symptoms in children [53]. Parental mental stress is also well associated with the exacerbation of asthma symptoms in their children [54, 55]. The probable mechanism behind stress and asthma symptoms is that stress enhances inflammatory responses (49), affecting the hypothalamic-pituitary-adrenal axis and the sympathetic-adrenal-medullary axis (49) and eventually exacerbating the condition. However, caregivers’ perception of neighborhood safety, even without any measurable stress, can be an independent risk for asthma symptoms to their children [18].

Our study also considered the exposure time of these two important neighborhood environmental factors (such as TRAP represented by heavy traffic on street; and neighborhood liveability scale), which were significantly associated with asthma symptom trajectories. Although most of the prior studies reported their association between TRAP and the onset of asthma symptoms, very few showed their impact on the trajectories of asthma symptoms in the longitudinal model [52, 56, 57]. The findings of our study revealed that other than persistent exposure to these factors, exposure in the first few years (0–5 years) of life was also associated with persistently high prevalence of asthma symptom trajectory throughout all of childhood. Therefore, early life exposure to these detrimental environmental exposures is a significant risk for having an unfavorable trajectory of asthma symptom (such as wheezing) during childhood as it is a crucial time for both structural and functional development of lungs [29, 31].

In our study, we also looked for the association between neighborhood facility score, general condition of nearby buildings, and neighborhood greenspace. Other than the neighborhood facility score, none had any significant association with asthma symptom trajectories in any analytical model. We considered green space as one of our exposure variables because several prior studies emphasized the amount of greenspace in the neighborhood and its protective function on childhood asthma [58–60]. However, a systematic review and meta-analysis could not find concrete evidence favoring this [61, 62]. One study from the same cohort (the LSAC study) reported that greenspace had no individual association with asthma symptom; however, it may have a protective effect on children suffering from asthma symptom by buffering traffic-related air pollution [17]. Our study also did not find any statistically significant association between neighborhood green space (considered as the average NDVI value of that area) and asthma symptom trajectories in cross-sectional models. Due to the nature of LSAC data, we considered the mean NDVI values for a specific postcode area as the condition of neighborhood greenspace. However, if we could measure the NDVI values of smaller spatial distribution, the results might be more precise.

## Strengths and limitations

The main strength of our study is that we used the data of a nationally representative sample of Australian children, with a longitudinal follow-up for asthma symptom and the presence of some neighborhood environmental factors. The use of a meticulous and well-established analysis technique also provided reliable results.

However, the study has several limitations. The main limitations of our study were missing values and participant attrition. We could not include all participants in our study due to a lack of information for our outcome variables. In our study, the analytic sample was significantly different in terms of some baseline characteristics from the participants who could not be included in the longitudinal analyses. Thus, it might create a selection bias. To address this limitation, we did sensitivity analyses of all our regression models after incorporating the IPW technique [44] to control selection bias [43]. Moreover, for the missing values of baseline characteristics and some independent variables in our analytic sample, we used multiple imputations (MI). Due to the lack of information in the LSAC study, we could not measure the direct associations between some hazardous neighborhood environmental factors, such as some gases from TRAP which were much highlighted in several prior studies in Australia [63]. However, the proxy indicator (heavy traffic on neighborhood streets) of TRAP in our study may give an overall impression of its trajectories and association with asthma symptom trajectories from a nation-wide data.

## Conclusion

In conclusion, the prevalence of several potential neighborhood environmental factors has not been remarkably changed over the last 15 years. However, neighborhood heavy traffic conditions and neighborhood liveability scale showed distinct trajectories. A significant proportion of children are exposed to relatively bad trajectories of these two environmental factors. Our study also found that children who had bad trajectories of these two environmental factors were highly associated with the transient and persistently high prevalence of asthma symptom trajectories throughout childhood. Moreover, these two environmental factors exerted a relatively higher risk of belonging to a bad trajectory of asthma symptom when exposed in the first few years of life. Our study’s findings provide substantial scientific evidence that has policy and practice implications for reducing asthma morbidity among children in Australia by taking adequate actions to minimize these exposures in childhood.

## Electronic supplementary material

Below is the link to the electronic supplementary material.

Supplementary Material 1
