# Supplementary material for: Trajectories of neighborhood environmental factors and their associations with asthma symptom trajectories among children in Australia: evidence from a national birth cohort study
Source: J Environ Health Sci Eng. 2022 Sep 13;20(2):835–47. doi: 10.1007/s40201-022-00824-z (PMC9672149; doi:10.1007/s40201-022-00824-z)
Supplement: Supplementary file 1 — Supplementary Material 1 [file 40201_2022_824_MOESM1_ESM.pdf]

**Supplemental Texts:**

Text ST1: Methodological approach for measuring Greenspace as Normalized Difference Vegetation Index (NDVI)

Text ST2: Methodological approach for Group-based trajectory modelling (GBTM)

**Supplemental Tables:**

Table S1: List of exposure variables

Table S2: List of the adjusting variables

Table S3: Selection criteria of the best-fitting model for 'heavy traffic on street' trajectories (n=4174)

Table S4: Selection criteria of the best-fitting model for neighbourhood liveability scale trajectories (n=4174)

Table S5: Sensitivity analysis between the complete case and imputed data on the multivariable association of neighbourhood environmental factors with asthma-symptom trajectories (Model 1)

Table S6: Sensitivity analysis between the complete case and imputed data on the multivariable association of neighbourhood environmental factors with asthma-symptom trajectories (Model 2)

Table S7: Sensitivity analysis between the complete case and imputed data on the multivariable association of neighbourhood environmental factors with asthma-symptom trajectories (Model 3)

Table S8: Sensitivity analysis before and after incorporating inverse probability weighting (IPW) technique (Multivariable model 1)

Table S9: Sensitivity analysis before and after incorporating inverse probability weighting (IPW) technique (Multivariable model 2)

Table S10: Sensitivity analysis before and after incorporating inverse probability weighting (IPW) technique (Multivariable model 3)

Table S11: Baseline characteristics of the analytical cohort and rest of the participants (N=5107)

Table S12: Prevalence (in %) of asthma-symptom and different neighbourhood environmental exposures over different time points among the cohort

Table S13a: Bivariate analyses showing the association (risk ratio, 95% CI) between different neighbourhood environmental exposures and asthma-symptom trajectories ('low' vs. 'transient high' groups) over different time points

Table S13b: Bivariate analyses showing the association (risk ratio, 95% CI) between different neighbourhood environmental exposures and asthma-symptom trajectories ('low' vs. 'persistent high' groups) over different time points

Table S14: Number and percentage of participants having bad trajectories of both heavy traffic on street and neighbourhood liveability score.

**Text ST1: Methodological approach for measuring Greenspace as Normalized Difference Vegetation Index (NDVI)**

We used data from 'The Bureau of Meteorology', Australia (http://www.bom.gov.au/climate/austmaps/about-ndvi-maps.shtml ) for Greenspace data. Normalized Difference Vegetation Index (NDVI) is a method of quantification of vegetation by measuring the difference between near-infrared (NIR) (which vegetation strongly reflects) and red light (which vegetation absorbs).

The NDVI is calculated from these individual measurements as follows:

$$NVDI = \frac{(NIR - Red)}{(NIR + Red)}$$

Its value is always between -1 and +1. Vegetation NDVI in Australia typically ranges from 0.1 up to 0.7, with higher values associated with greater density and greenness of the plant canopy. NDVI decreases as leaves come under water stress, become diseased or die. Bare soil and snow values are close to zero, while water bodies have negative values. Further details about NDVI can be obtained from the bureau's website ( http://www.bom.gov.au/climate/austmaps/about-ndvi-maps.shtml )

For our study, we collected 6 monthly NDVI average (January- June, and July -December) for 2004-2018 from the BOM website in grid format. We also collected digital boundary map of Australia as 'Shapefile' from 'Australian Bureau of Statistics' (https://www.abs.gov.au/statistics/standards/australian-statistical-geography-standard-asgs-edition-3/jul2021-jun2026/access-and-downloads/digital-boundary-files ). Then by using ArcGIS software (ArcGIS Pro, version 2.6.2, 2020 Esri Inc) we plotted the NDVI grid file on the digital boundary map of Australia (Shapefile) and calculated the average NDVI values of a specific area (we used Shapefile for 'Postal Areas' as LSAC study used 'Postal Areas' as sampling frames). Although BOM provided NDVI data as six monthlies average, we calculated the biannual average from six monthlies average as LSAC collected data from the participants biannually.

**Text ST2: Methodological approach for Group-based trajectory modelling (GBTM)**

GBTM is a specialized form of finite mixture modelling, designed to identify groups of individuals following similar developmental trajectories.[1,2] Unlike hierarchical and latent class modelling, GBTM does not require parameters to be continuously distributed throughout the population according to the multivariate normal distribution. This offers the ability to classify children who belong to a distinct disease trajectory with group-specific time-related parameters and explore common risk factors and environmental exposures that may impact each identified group.[3] The model was repeatedly fit in a stepwise manner to choose the appropriate number of trajectory groups, starting with two groups. GBTM does not identify the true number of trajectory groups because there is no true number. Instead, it identifies the distinctive features of the data.[4] In our study, a total of 79 different GBTM models were generated by using a different number of trajectory groups, polynomial distribution, and orders. All models were then ranked as per their Bayesian Information Criteria (BIC) and Akaike information criterion (AIC). Based on relatively lowest BIC and AIC, the top 3-5 models were further evaluated for (i) the sample size in each group (a minimum of 7% of the total sample); (ii) the statistical significance of the polynomial coefficients; and (iii) a close correspondence between the estimated probability of group membership presented as an average posterior probability (>0.7).[2,5] More detailed information on polynomial distributions and selection of the best model are presented in Table S3-4.

**Table S1: List of exposure variables**

| | Topic | Variables | Response |
|---|---|---|---|
| 1 | Traffic condition on street | Heavy traffic on street | Agree, disagree |
| 2 | Neighbourhood housing | General condition of nearby buildings within 100 metres of the respondent's house | Good, bad |
| 3 | Neighbourhood liveability scale | Scale based on the mean of responses (1 Strongly agree; 2 Agree; 3 Neither disagree nor agree; 4 Disagree; 5 Strongly disagree) for the following question:<br><br>I. This is a safe neighbourhood<br>II. There are good parks, playgrounds and play spaces in this neighbourhood<br>III. The state of footpaths, roads and street lighting is good in this neighbourhood | Number<br><br>(Lower number in the scale indicates better condition) |
| 4 | Neighbourhood facilities scale | Scale based on the mean of responses (1 Strongly agree; 2 Agree; 3 Neither disagree nor agree; 4 Disagree; 5 Strongly disagree) for the following question:<br><br>I. There is access to close, affordable, regular public transport in this neighbourhood<br>II. There is access to basic shopping facilities in this neighbourhood.<br>III. There is access to basic services such as banks, medical clinics, etc. in this neighbourhood | Number<br><br>(Lower number in the scale indicates better condition) |
| 5 | Neighbourhood greenspace | Average Normalized Difference Vegetation Index (NDVI) value of that area for a specific year | This index defines values from -1.0 to 1.0.<br><br>(Higher value indicates better greenspace) |

**Table S2: List of the adjusting variables**

| | Topic | variables | Response |
|---|---|---|---|
| 1 | Sex | Sex of the child | Male, female |
| 2 | Indigenous status | From Indigenous background? | Yes, no |
| 3 | Health status | Any current health condition | Yes, no |
| 4 | Pre-term birth | Pre-term birth | Yes, no |
| 5 | Current medication for asthma | Current medication for asthma | Yes, no |
| 6 | Maternal asthma medication during pregnancy | Maternal asthma medication during pregnancy | Yes, no |
| 7 | Smoking in pregnancy | Maternal cigarette smoking during pregnancy | Yes, no |
| 8 | Socioeconomic status | Socio-Economic Indexes for Areas (SEIFA) economic resources | Number |

**Table S3: Selection criteria of the best-fitting model for 'heavy traffic on street' trajectories (n=4174)**

S3a)

| SL | Group number | Polynomial distribution | BIC€ | AIC¥ | BIC rank | AIC rank |
|---|---|---|---|---|---|---|
| 1 | 2 | 0 0 | 14541.18 | 14528.74 | 58 | 58 |
| 2 | 2 | 0 1 | 14546.18 | 14529.59 | 60 | 60 |
| 3 | 2 | 0 2 | 14551.31 | 14530.57 | 63 | 65 |
| 4 | 2 | 0 3 | 14556.13 | 14531.24 | 67 | 68 |
| 5 | 2 | 1 0 | 14545.88 | 14529.29 | 59 | 59 |
| 6 | 2 | 1 1 | 14550.79 | 14530.05 | 62 | 62 |
| 7 | 2 | 1 2 | 14555.91 | 14531.03 | 66 | 66 |
| 8 | 2 | 1 3 | 14560.74 | 14531.71 | 70 | 70 |
| 9 | 2 | 2 0 | 14550.35 | 14529.62 | 61 | 61 |
| 10 | 2 | 2 1 | 14555.25 | 14530.37 | 65 | 64 |
| 11 | 2 | 2 2 | 14560.40 | 14531.37 | 69 | 69 |
| 12 | 2 | 2 3 | 14565.21 | 14532.04 | 71 | 71 |
| 13 | 2 | 3 0 | 14555.18 | 14530.30 | 64 | 63 |
| 14 | 2 | 3 1 | 14560.08 | 14531.05 | 68 | 67 |
| 15 | 2 | 3 2 | 14565.22 | 14532.05 | 72 | 72 |
| 16 | 2 | 3 3 | 14569.89 | 14532.57 | 73 | 73 |
| 17 | 3 | **0 1 0** | **14346.38** | **14321.49** | **1** | **2** |
| 18 | 3 | 0 1 1 | 14350.93 | 14321.90 | 4 | 5 |
| 19 | 3 | 0 1 2 | 14356.01 | 14322.83 | 12 | 15 |
| 20 | 3 | 0 1 3 | 14361.04 | 14323.71 | 25 | 30 |
| 21 | 3 | 0 2 0 | 14351.33 | 14322.30 | 5 | 9 |
| 22 | 3 | 0 2 1 | 14355.88 | 14322.71 | 11 | 14 |
| 23 | 3 | 0 2 2 | 14361.00 | 14323.68 | 23 | 28 |
| 24 | 3 | 0 2 3 | 14366.02 | 14324.55 | 36 | 40 |
| 25 | 3 | 0 3 0 | 14356.46 | 14323.28 | 13 | 21 |

| | | | | | | |
|---|---|---|---|---|---|---|
| 26 | 3 | 0 3 1 | 14361.01 | 14323.69 | 24 | 29 |
| 27 | 3 | 0 3 2 | 14366.13 | 14324.66 | 37 | 43 |
| 28 | 3 | 0 3 3 | 14371.16 | 14325.55 | 47 | 52 |
| 29 | 3 | **1 0 1** | **14350.26** | **14321.23** | **2** | **1** |
| 30 | 3 | 1 0 2 | 14355.34 | 14322.16 | 8 | 7 |
| 31 | 3 | 1 0 3 | 14360.36 | 14323.04 | 19 | 18 |
| 32 | 3 | **1 1 0** | **14350.74** | **14321.71** | **3** | **4** |
| 33 | 3 | 1 1 1 | 14355.38 | 14322.20 | 9 | 8 |
| 34 | 3 | 1 1 2 | 14360.46 | 14323.13 | 21 | 20 |
| 35 | 3 | 1 1 3 | 14365.48 | 14324.01 | 34 | 34 |
| 36 | 3 | 1 2 0 | 14355.69 | 14322.52 | 10 | 11 |
| 37 | 3 | 1 2 1 | 14360.33 | 14323.01 | 18 | 17 |
| 38 | 3 | 1 2 2 | 14365.45 | 14323.98 | 32 | 32 |
| 39 | 3 | 1 2 3 | 14370.47 | 14324.85 | 44 | 44 |
| 40 | 3 | 1 3 0 | 14360.83 | 14323.50 | 22 | 25 |
| 41 | 3 | 1 3 1 | 14365.47 | 14324.00 | 33 | 33 |
| 42 | 3 | 1 3 2 | 14370.58 | 14324.97 | 46 | 46 |
| 43 | 3 | 1 3 3 | 14375.61 | 14325.85 | 53 | 53 |
| 44 | 3 | 2 0 1 | 14354.86 | 14321.68 | 6 | 3 |
| 45 | 3 | 2 0 2 | 14359.94 | 14322.62 | 15 | 12 |
| 46 | 3 | 2 0 3 | 14364.96 | 14323.49 | 28 | 24 |
| 47 | 3 | 2 1 0 | 14355.33 | 14322.15 | 7 | 6 |
| 48 | 3 | 2 1 1 | 14359.97 | 14322.65 | 16 | 13 |
| 49 | 3 | 2 1 2 | 14365.05 | 14323.58 | 29 | 26 |
| 50 | 3 | 2 1 3 | 14370.08 | 14324.46 | 41 | 39 |
| 51 | 3 | 2 2 0 | 14360.42 | 14323.10 | 20 | 19 |
| 52 | 3 | 2 2 1 | 14365.07 | 14323.60 | 30 | 27 |
| 53 | 3 | 2 2 2 | 14370.17 | 14324.55 | 42 | 41 |
| 54 | 3 | 2 2 3 | 14375.19 | 14325.42 | 51 | 50 |
| 55 | 3 | 2 3 0 | 14365.56 | 14324.09 | 35 | 35 |

| | | | | | | |
|---|---|---|---|---|---|---|
| 56 | 3 | 2 3 1 | 14370.20 | 14324.58 | 43 | 42 |
| 57 | 3 | 2 3 2 | 14375.30 | 14325.54 | 52 | 51 |
| 58 | 3 | 2 3 3 | 14380.33 | 14326.42 | 56 | 56 |
| 59 | 3 | 3 0 1 | 14359.82 | 14322.50 | 14 | 10 |
| 60 | 3 | 3 0 2 | 14364.90 | 14323.43 | 26 | 22 |
| 61 | 3 | 3 0 3 | 14369.93 | 14324.31 | 38 | 36 |
| 62 | 3 | 3 1 0 | 14360.29 | 14322.97 | 17 | 16 |
| 63 | 3 | 3 1 1 | 14364.94 | 14323.47 | 27 | 23 |
| 64 | 3 | 3 1 2 | 14370.02 | 14324.40 | 39 | 37 |
| 65 | 3 | 3 1 3 | 14375.04 | 14325.28 | 48 | 47 |
| 66 | 3 | 3 2 0 | 14365.39 | 14323.92 | 31 | 31 |
| 67 | 3 | 3 2 1 | 14370.03 | 14324.42 | 40 | 38 |
| 68 | 3 | 3 2 2 | 14375.14 | 14325.37 | 50 | 49 |
| 69 | 3 | 3 2 3 | 14380.16 | 14326.25 | 54 | 54 |
| 70 | 3 | 3 3 0 | 14370.47 | 14324.86 | 45 | 45 |
| 71 | 3 | 3 3 1 | 14375.12 | 14325.35 | 49 | 48 |
| 72 | 3 | 3 3 2 | 14380.22 | 14326.30 | 55 | 55 |
| 73 | 3 | 3 3 3 | 14385.27 | 14327.22 | 57 | 57 |
| 74 | 4 | 0 0 0 1 | 14342.31 | 14309.13 | One group has <7% participants | |
| 75 | 4 | 0 0 1 1 | 14347.45 | 14310.13 | One group has <7% participants | |
| 76 | 4 | 0 0 1 2 | 14352.59 | 14311.12 | One group has <7% participants | |
| 77 | 4 | 0 0 2 2 | 14357.67 | 14312.05 | One group has <7% participants | |
| 78 | 4 | 0 1 1 1 | 14352.44 | 14310.97 | One group has <7% participants | |
| 79 | 4 | 0 2 2 2 | 14367.29 | 14313.38 | One group has <7% participants | |

S3b)

| Polynomial distribution | BIC[€] | AIC[¥] | Group% in model | | | APP[£] | | | p-value | | |
|---|---|---|---|---|---|---|---|---|---|---|---|
| | | | [©]*G1* | *G2* | *G3* | *G1* | *G2* | *G3* | *G1* | *G2* | *G3* |
| **0 1 0** | **14346.38** | **14321.49** | **56** | **34** | **9** | **0.91** | **0.82** | **0.82** | **0.571** | **0.333** | **0.095** |
| 1 0 1 | 14350.26 | 14321.23 | 56 | 34 | 9 | 0.91 | 0.81 | 0.84 | 0.568 | 0.342 | 0.089 |
| 1 1 0 | 14350.74 | 14321.71 | 56 | 34 | 9 | 0.91 | 0.81 | 0.85 | 0.567 | 0.345 | 0.086 |

BIC[€]= Bayesian information criterion; AIC[¥]= Akaike information criterion; APP[£]= average posterior probability; G[©]= group

**Summary**: All four groups polynomial distribution showed one of the groups had <7% participants. However, a model of three groups polynomial distribution (0 1 0) had best fitting model with relatively lower BIC and AIC values, none of their group's app value below 0.7, p-value >0.05, and none of their group's membership was below 7%

**Table S4: Selection criteria of the best-fitting model for neighbourhood liveability scale trajectories (n=4174)**

(S4a)

| SL | Group number | Polynomial distribution | BIC[€] | AIC[¥] | BIC rank | AIC rank |
|---|---|---|---|---|---|---|
| 1 | 2 | 0 0 | 27956.72 | 27940.13 | 79 | 79 |
| 2 | 2 | 0 1 | 27885.98 | 27865.24 | 78 | 78 |
| 3 | 2 | 0 2 | 27835.36 | 27810.47 | 76 | 77 |
| 4 | 2 | 0 3 | 27835.49 | 27806.45 | 77 | 76 |
| 5 | 2 | 1 0 | 27386.67 | 27365.93 | 75 | 75 |
| 6 | 2 | 1 1 | 27352.01 | 27327.12 | 72 | 72 |
| 7 | 2 | 1 2 | 27305.74 | 27276.71 | 69 | 69 |
| 8 | 2 | 1 3 | 27304.66 | 27271.47 | 68 | 68 |
| 9 | 2 | 2 0 | 27373.09 | 27348.20 | 73 | 73 |
| 10 | 2 | 2 1 | 27337.24 | 27308.21 | 70 | 70 |
| 11 | 2 | **2 2** | **27284.62** | **27251.44** | **65** | **66** |

| | | | | | | |
|---|---|---|---|---|---|---|
| 12 | 2 | **2 3** | **27283.27** | **27245.94** | **64** | **65** |
| 13 | 2 | 3 0 | 27377.48 | 27348.44 | 74 | 74 |
| 14 | 2 | 3 1 | 27341.65 | 27308.47 | 71 | 71 |
| 15 | 2 | 3 2 | 27288.91 | 27251.58 | 67 | 67 |
| 16 | 2 | **3 3** | **27287.13** | **27245.65** | **66** | **64** |
| 17 | 3 | 0 1 0 | 26822.77 | 26793.73 | 58 | 62 |
| 18 | 3 | 0 1 1 | 26826.14 | 26792.95 | 61 | 61 |
| 19 | 3 | 0 1 2 | 26731.27 | 26693.94 | 51 | 56 |
| 20 | 3 | 0 1 3 | 26732.90 | 26691.42 | 52 | 54 |
| 21 | 3 | 0 2 0 | 26821.30 | 26788.11 | 57 | 60 |
| 22 | 3 | 0 2 1 | 26824.34 | 26787.01 | 60 | 59 |
| 23 | 3 | 0 2 2 | 26733.79 | 26692.31 | 53 | 55 |
| 24 | 3 | 0 2 3 | 26735.57 | 26689.94 | 54 | 52 |
| 25 | 3 | 0 3 0 | 26824.01 | 26786.68 | 59 | 58 |
| 26 | 3 | 0 3 1 | 26827.13 | 26785.65 | 62 | 57 |
| 27 | 3 | 0 3 2 | 26736.88 | 26691.25 | 55 | 53 |
| 28 | 3 | 0 3 3 | 26739.07 | 26689.29 | 56 | 51 |
| 29 | 3 | 1 0 1 | 26402.03 | 26368.85 | 50 | 50 |
| 30 | 3 | 1 0 2 | 26307.84 | 26270.51 | 46 | 47 |
| 31 | 3 | 1 0 3 | 26310.14 | 26268.66 | 47 | 46 |
| 32 | 3 | 1 1 0 | 26271.95 | 26238.76 | 38 | 41 |
| 33 | 3 | 1 1 1 | 26275.41 | 26238.07 | 41 | 40 |
| 34 | 3 | 1 1 2 | 26178.70 | 26137.22 | 17 | 22 |
| 35 | 3 | 1 1 3 | 26180.52 | 26134.89 | 18 | 20 |
| 36 | 3 | 1 2 0 | 26270.51 | 26233.18 | 36 | 39 |
| 37 | 3 | 1 2 1 | 26273.75 | 26232.27 | 39 | 38 |
| 38 | 3 | 1 2 2 | 26181.23 | 26135.60 | 19 | 21 |
| 39 | 3 | 1 2 3 | 26183.16 | 26133.38 | 21 | 19 |
| 40 | 3 | 1 3 0 | 26271.61 | 26230.13 | 37 | 37 |
| 41 | 3 | 1 3 1 | 26274.96 | 26229.33 | 40 | 36 |

| | | | | | | |
|---|---|---|---|---|---|---|
| 42 | 3 | 1 3 2 | 26182.74 | 26132.97 | 20 | 18 |
| 43 | 3 | 1 3 3 | 26185.17 | 26131.25 | 22 | 17 |
| 44 | 3 | 2 0 1 | 26390.04 | 26352.71 | 48 | 49 |
| 45 | 3 | 2 0 2 | 26295.68 | 26254.20 | 42 | 45 |
| 46 | 3 | 2 0 3 | 26297.97 | 26252.34 | 43 | 43 |
| 47 | 3 | 2 1 0 | 26253.93 | 26216.60 | 30 | 35 |
| 48 | 3 | 2 1 1 | 26257.34 | 26215.86 | 34 | 34 |
| 49 | 3 | 2 1 2 | 26160.29 | 26114.66 | 5 | 16 |
| 50 | 3 | 2 1 3 | 26162.09 | 26112.31 | 7 | 14 |
| 51 | 3 | 2 2 0 | 26249.52 | 26208.04 | 24 | 31 |
| 52 | 3 | 2 2 1 | 26252.68 | 26207.05 | 28 | 30 |
| 53 | 3 | 2 2 2 | 26160.81 | 26111.04 | 6 | 13 |
| 54 | 3 | 2 2 3 | 26162.76 | 26108.83 | 11 | 11 |
| 55 | 3 | 2 3 0 | 26250.39 | 26204.76 | 25 | 28 |
| 56 | 3 | 2 3 1 | 26253.66 | 26203.89 | 29 | 25 |
| 57 | 3 | 2 3 2 | 26162.10 | 26108.18 | 8 | 9 |
| 58 | 3 | 2 3 3 | 26164.56 | 26106.48 | 14 | 7 |
| 59 | 3 | 3 0 1 | 26392.61 | 26351.13 | 49 | 48 |
| 60 | 3 | 3 0 2 | 26298.21 | 26252.58 | 44 | 44 |
| 61 | 3 | 3 0 3 | 26300.50 | 26250.72 | 45 | 42 |
| 62 | 3 | 3 1 0 | 26255.91 | 26214.43 | 33 | 33 |
| 63 | 3 | 3 1 1 | 26259.37 | 26213.74 | 35 | 32 |
| 64 | 3 | 3 1 2 | 26162.28 | 26112.50 | 9 | 15 |
| 65 | 3 | 3 1 3 | 26164.07 | 26110.15 | 13 | 12 |
| 66 | 3 | 3 2 0 | 26251.31 | 26205.68 | 26 | 29 |
| 67 | 3 | 3 2 1 | 26254.52 | 26204.74 | 31 | 27 |
| 68 | 3 | 3 2 2 | 26162.67 | 26108.75 | 10 | 10 |
| 69 | 3 | 3 2 3 | 26164.62 | 26106.54 | 15 | 8 |
| 70 | 3 | 3 3 0 | 26251.40 | 26201.63 | 27 | 24 |
| 71 | 3 | 3 3 1 | 26254.74 | 26200.82 | 32 | 23 |

| | | | | | | |
|---|---|---|---|---|---|---|
| 72 | 3 | 3 3 2 | 26163.23 | 26105.15 | 12 | 6 |
| 73 | 3 | 3 3 3 | 26165.73 | 26103.51 | 16 | 4 |
| 74 | 4 | 0 0 0 1 | 26835.59 | 26798.26 | 63 | 63 |
| 75 | 4 | 0 0 1 1 | 26245.62 | 26204.14 | 23 | 26 |
| 76 | 4 | 0 0 1 2 | 26149.59 | 26103.97 | 4 | 5 |
| 77 | 4 | 0 0 2 2 | 26144.57 | 26094.79 | 3 | 3 |
| 78 | 4 | 0 1 1 1 | 26074.93 | 26029.3 | 2 | 2 |
| 79 | 4 | 0 2 2 2 | 26054.33 | 25996.26 | 1 | 1 |

S4b)

| **Polynomial distribution** | **BIC[€]** | **AIC[¥]** | **Group% in model** | | | **APP[£]** | | | **p-value** | | |
|---|---|---|---|---|---|---|---|---|---|---|---|
| | | | [©]***G1*** | ***G2*** | ***G3*** | ***G1*** | ***G2*** | ***G3*** | ***G1*** | ***G2*** | ***G3*** |
| **2 3** | 27283.27 | 27245.94 | **35** | **64** | **x** | **0.92** | **0.94** | **x** | **0.343** | **0.656** | **x** |
| 3 3 | 27287.13 | 27245.65 | 35 | 64 | x | 0.92 | 0.94 | x | 0.342 | 0.657 | x |
| 2 2 | 27284.62 | 27251.44 | 35 | 65 | x | 0.92 | 0.94 | x | 0.342 | 0.657 | x |

BIC[€]= Bayesian information criterion; AIC[¥]= Akaike information criterion; APP[£]= average posterior probability; G[©]= group

**Summary**: One group have <7% participants in all four groups model. Although three groups models had less AIC and BIC values, two trajectories groups in these models showed less difference from each other. So, we considered two groups model, and one 'two groups polynomial distribution (2 3) had best fitting model with relatively lowest BIC and AIC values (among all the two groups models), comparative group's app values, and none of their group's membership was below 7%.

**Table S5: Sensitivity analysis between the complete case and imputed data on the multivariable association of neighbourhood environmental factors with asthma-symptom trajectories (Model 1)**

| Variables | Complete case analysis (N= 3584) | | Imputed data set (N =4174) | |
|---|---|---|---|---|
| | Transient high | Persistent high | Transient high | Persistent high |
| Heavy traffic on street | | | | |
| Disagree | 1 | 1 | 1 | 1 |
| Agree | 1.11(0.85,1.45) | 1.25(0.98,1.60) | 1.13(0.90,1.43) | 1.31(1.07,1.62) |
| General condition of nearby buildings | | | | |
| Good | 1 | 1 | 1 | 1 |
| Bad | 1.60(0.66,3.86) | 0.36(0.10,1.30) | 0.81(0.34,1.87) | 0.41(0.15,1.09) |
| Neighbourhood liveability scale | 1.28(0.98,1.66) | 1.44(1.13,1.84) | 1.15(0.92,1.43) | 1.33(1.08,1.63) |
| Neighbourhood facilities scale | 0.94(0.78,1.12) | 1.12(0.95,1.32) | 1.01(0.87,1.16) | 1.01(0.88,1.16) |
| NDVI value of the area | 1.45(0.51,4.10) | 0.45(0.17,1.20) | 1.17(0.47,2.89) | 0.54(0.23,1.26) |

Association presented as risk ratio and their 95% confidence interval, 1= Reference value; Reference group for Trajectory is 'No/low asthma-symptom trajectory group'. Adjusted for sex, Indigenous status, SEIFA economic resources, any medical condition, pre-term birth, asthma medication of mother during pregnancy, maternal smoking during pregnancy.

**Table S6: Sensitivity analysis between the complete case and imputed data on the multivariable association of neighbourhood environmental factors with asthma-symptom trajectories (Model 2)**

| Variables | | Complete case analysis (N= 4146) | | Imputed data set (N =4174) | |
|---|---|---|---|---|---|
| | Group % | Transient high | Persistent high | Transient high | Persistent high |
| Heavy traffic on street_ agree | | | | | |
| Never | 55% | 1 | 1 | 1 | 1 |
| 1-2 time points | 37% | 1.27(1.17,1.39) | 1.16(1.06,1.27) | 1.28(1.19,1.38) | 1.24(1.15,1.34) |
| All 3 time points | 8% | 1.25(1.07,1.46) | 1.32(1.13,1.54) | 1.35(1.18,1.54) | 1.47(1.29,1.68) |
| General condition of nearby buildings_ bad | | | | | |
| Never | 97% | 1 | 1 | 1 | 1 |
| 1-2 time points | 3% | 1.21(0.96,1.53) | 0.87(0.67,1.12) | 0.93(0.76,1.14) | 0.85(0.69,1.04) |
| All 3 time points | <1% | Not done* | Not done* | Not done | Not done |
| Neighbourhood liveability scale (mean of 3 time points) | | 0.99(0.88,1.10) | 1.30(1.16,1.46) | 0.97(0.88,1.07) | 1.37(1.24,1.51) |
| Neighbourhood facilities scale (mean of 3 time points) | | 1.21(1.12,1.30) | 1.09(1.00,1.18) | 1.16(1.08,1.23) | 1.05(0.98,1.12) |
| NDVI value of the area (mean of 3 time points) | | 1.24(0.82,1.88) | 0.77(0.50,1.18) | 1.12(0.78,1.60) | 0.81(0.56,1.17) |

Association presented as risk ratio and their 95% confidence interval, 1= Reference value; Reference group for Trajectory is 'No/low asthma-symptom trajectory group'. Adjusted for sex, Indigenous status, SEIFA economic resources, any medical condition, pre-term birth, asthma medication of mother during pregnancy, maternal smoking during pregnancy, asthma medication.

**Table S7: Sensitivity analysis between the complete case and imputed data on the multivariable association of neighbourhood environmental factors with asthma-symptom trajectories (Model 3)**

| Variables | Complete case analysis (N=4143) | | Imputed data set (N =4174) | |
|---|---|---|---|---|
| | Transient high | Persistent high | Transient high | Persistent high |
| **Prevalence of heavy traffic on street (trajectory groups)** | | | | |
| Low | 1 | 1 | 1 | 1 |
| Persistently moderate | 1.16(1.07,1.27) | 1.21(1.10,1.32) | 1.21(1.12,1.31) | 1.29(1.20,1.40) |
| Persistently high | 1.37(1.20,1.56) | 1.10(0.95,1.28) | 1.40(1.25,1.58) | 1.33(1.17,1.50) |
| **Neighbourhood liveability score (trajectory groups)** | | | | |
| Low & declining | 1 | 1 | 1 | 1 |
| Moderate & static | 1.20(1.09,1.31) | 1.42(1.30,1.56) | 1.16(1.07,1.25) | 1.38(1.27,1.50) |

Association presented as risk ratio and their 95% confidence interval, 1= Reference value; Reference group for Trajectory is 'No/low asthma-symptom trajectory group'. Adjusted for sex, Indigenous status, SEIFA economic resources, any medical condition, pre-term birth, asthma medication of mother during pregnancy, maternal smoking during pregnancy, asthma medication.

**Table S8: Sensitivity analysis before and after incorporating inverse probability weighting (IPW) technique (Multivariable model 1)**

| Variables | Before IPW (N=4174) | | After IPW (N=4174) | |
|---|---|---|---|---|
| | Transient high | Persistent high | Transient high | Persistent high |
| Heavy traffic on street | | | | |
| Disagree | 1 | 1 | 1 | 1 |
| Agree | 1.13(0.90,1.43) | 1.31(1.07,1.62) | 1.12(0.89,1.42) | 1.33(1.08,1.64) |
| General condition of nearby buildings | | | | |
| Good | 1 | 1 | 1 | 1 |
| Bad | 0.81(0.34,1.87) | 0.41(0.15,1.09) | 0.75(0.30,1.86) | 0.38(0.14,1.01) |
| Neighbourhood liveability scale | 1.15(0.92,1.43) | 1.33(1.08,1.63) | 1.14(0.91,1.43) | 1.32(1.06,1.63) |
| Neighbourhood facilities scale | 1.01(0.87,1.16) | 1.01(0.88,1.16) | 1.01(0.87,1.18) | 1.01(0.88,1.15) |
| NDVI value of the area | 1.17(0.47,2.89) | 0.54(0.23,1.26) | 1.16(0.47,2.84) | 0.53(0.23,1.22) |

Association presented as risk ratio and their 95% confidence interval, 1= Reference value; Reference group for Trajectory is 'No/low asthma-symptom trajectory group'. Adjusted for sex, Indigenous status, SEIFA economic resources, any medical condition, pre-term birth, asthma medication of mother during pregnancy, maternal smoking during pregnancy.

**Table S9: Sensitivity analysis before and after incorporating inverse probability weighting (IPW) technique (Multivariable model 2)**

| Variables | | Before IPW (N=4174) | | After IPW (N=4174) | |
|---|---|---|---|---|---|
| | Group % | Transient high | Persistent high | Transient high | Persistent high |
| Heavy traffic on street_ agree | | | | | |
| Never | 55% | 1 | 1 | 1 | 1 |
| 1-2 time points | 37% | 1.28(1.19,1.38) | 1.24(1.15,1.34) | 1.27(1.18,1.38) | 1.25(1.16,1.35) |
| All 3 time points | 8% | 1.35(1.18,1.54) | 1.47(1.29,1.68) | 1.31(1.15,1.50) | 1.47(1.30,1.67) |
| General condition of nearby buildings_ bad | | | | | |
| Never | 97% | 1 | 1 | 1 | 1 |
| 1-2 time points | 3% | 0.93(0.76,1.14) | 0.85(0.69,1.04) | 0.93(0.76,1.13) | 0.85(0.69,1.04) |
| All 3 time points | <1% | Not done | Not done | Not done | Not done |
| Neighbourhood liveability scale (mean of 3 time points) | | 0.97(0.88,1.07) | 1.37(1.24,1.51) | 0.98(0.89,1.08) | 1.35(1.22,1.49) |
| Neighbourhood facilities scale (mean of 3 time points) | | 1.16(1.08,1.23) | 1.05(0.98,1.12) | 1.15(1.07,1.23) | 1.04(0.97,1.11) |
| NDVI value of the area (mean of 3 time points) | | 1.12(0.78,1.60) | 0.81(0.56,1.17) | 1.12(0.78,1.60) | 0.81(0.56,1.18) |

Association presented as risk ratio and their 95% confidence interval, 1= Reference value; Reference group for Trajectory is 'No/low asthma-symptom trajectory group'. Adjusted for sex, Indigenous status, SEIFA economic resources, any medical condition, pre-term birth, asthma medication of mother during pregnancy, maternal smoking during pregnancy, asthma medication.

**Table S10: Sensitivity analysis before and after incorporating inverse probability weighting (IPW) technique (Multivariable model 3)**

| Variables | Before IPW (N=4174) | | After IPW (N=4174) | |
|---|---|---|---|---|
| | Transient high | Persistent high | Transient high | Persistent high |
| **Prevalence of heavy traffic on street (trajectory groups)** | | | | |
| Low | 1 | 1 | 1 | 1 |
| Persistently moderate | 1.21(1.12,1.31) | 1.29(1.20,1.40) | 1.22(1.13,1.32) | 1.30(1.20,1.41) |
| Persistently high | 1.40(1.25,1.58) | 1.33(1.17,1.50) | 1.39(1.23,1.56) | 1.35(1.20,1.53) |
| **Neighbourhood liveability score (trajectory groups)** | | | | |
| Low & declining | 1 | 1 | 1 | 1 |
| Moderate & static | 1.16(1.07,1.25) | 1.38(1.27,1.50) | 1.16(1.07,1.25) | 1.37(1.26,1.49) |

Association presented as risk ratio and their 95% confidence interval, 1= Reference value; Reference group for Trajectory is 'No/low asthma-symptom trajectory group'; Adjusted for sex, Indigenous status, SEIFA economic resources, any medical condition, pre-term birth, asthma medication of mother during pregnancy, maternal smoking during pregnancy, asthma medication.

**Table S11: Baseline characteristics of the analytical cohort and rest of the participants (N=5107)**

| Variables | Analytic sample<br>*n= 4174 (82)* | Rest of the participants<br>*n=933 (18)* | *P* value |
|---|---|---|---|
| Male sex (n, %) | 2145(51) | 463(50) | 0.330 |
| Ethnicity- Indigenous (n, %) | 133(3) | 97(10) | **<0.001** |
| Low birth weight (n, %) | 214/4152(5) | 57/919(6) | 0.201 |
| Pre-term birth (n, %) | 262/4139(6) | 72/911(8) | 0.084 |
| Presence of any medical condition_ yes (n, %) | 216(5) | 67(7) | **0.015** |
| Maternal smoking during pregnancy_ yes (n, %) | 546(15) | 163(28) | **<0.001** |
| Asthma medication during pregnancy_ yes (n, %) | 150/4165(4) | 35(4) | 0.820 |
| SEIFA economic resources | | | |
| Lowest quantile | 1519(36) | 257(27) | **<0.001** |
| Second quantile | 847(20) | 244(26) | |
| Third quantile | 875(21) | 275(29) | |
| Highest quantile | 933(22) | 157(17) | |

SE = standard error; SEIFA= Socio-Economic Indexes for Areas

**Table S12: Prevalence (in %) of asthma-symptom and different neighbourhood environmental exposures over different time points among the cohort**

| Variable | Time points (age of the children) | | | | | | | |
|---|---|---|---|---|---|---|---|---|
| | **0-1 y** | **2-3 y** | **4-5 y** | **6-7 y** | **8-9 y** | **10-11 y** | **12-13 y** | **14-15 y** |
| Asthma-symptom (male, female) | 15.37 (17,13) | 24.52 (26,22) | 18.45 (19,17) | 17.66 (19,16) | 12.28 (14,10) | 10.48 (11.10) | 8.74 (10.8) | 12.59 (12,13) |
| Neighbourhood liveability scale | 2.01 | 1.99 | 1.95 | 1.85 | 2.07 | 1.82 | 1.77 | 1.79 |
| Neighbourhood facilities scale | 1.92 | 1.92 | 1.92 | 1.92 | 1.93 | 1.92 | 1.91 | 1.93 |
| Heavy traffic on street_ Yes | 25.55 | 27.20 | 25.86 | 25.60 | 26.64 | 25.82 | 25.37 | 24.87 |
| General condition of nearby buildings_ bad | 1.53 | 1.40 | 1.34 | 1.17 | 1.55 | 1.10 | 1.23 | 1.08 |
| NDVI value of the area | 0.34 | 0.34 | 0.33 | 0.33 | 0.34 | 0.33 | 0.33 | 0.33 |

**Table S13a: Bivariate analyses showing the association (risk ratio, 95% CI) between different neighbourhood environmental exposures and asthma-symptom trajectories (‘low’ vs. ‘transient high’ groups) over different time points**

| Variable | Time points (age of the children) | | | | | | | |
|---|---|---|---|---|---|---|---|---|
| | 0-1 y | 2-3 y | 4-5 y | 6-7 y | 8-9 y | 10-11 y | 12-13 y | 14-15 y |
| Heavy traffic on street_ Yes | 1.20 (0.96,1.50) | **1.24 (1.00,1.54)** | **1.36 (1.09,1.70)** | 1.23 (0.98,1.54) | **1.35 (1.08,1.68)** | **1.43 (1.14,1.81)** | **1.31 (1.02,1.69)** | 1.14 (0.87,1.50) |
| Neighbourhood liveability scale | **1.27 (1.03,1.56)** | 1.18 (0.98,1.42) | 1.02 (0.85,1.23) | 1.00 (0.84,1.20) | **1.20 (1.05,1.37)** | **1.25 (1.04,1.50)** | **1.30 (1.07,1.58)** | 1.21 (0.99,1.48) |
| Neighbourhood facilities scale | 1.07 (0.94,1.22) | 1.11 (0.97,1.27) | **1.17 (1.02,1.34)** | 1.04 (0.91,1.19) | 1.09 (0.96,1.25) | 1.12 (0.98,1.28) | 1.13 (0.97,1.32) | 1.15 (0.99,1.34) |
| General condition of nearby buildings_ bad | 1.34 (0.65,2.76) | 1.47 (0.68,3.18) | 1.65 (0.75,3.59) | **2.86 (1.40,5.81)** | 1.27 (0.59,2.73) | 1.32 (0.50,3.46) | **2.69 (1.19,6.11)** | 1.99 (0.73,5.38) |
| NDVI value of the area | 1.53 (0.66,3.54) | 1.18 (0.50,2.75) | 1.63 (0.70,3.82) | 1.80 (0.77,4.19) | 1.34 (0.57,3.15) | 1.49 (0.60,3.72) | 2.02 (0.78,5.23) | 1.03 (0.38,2.79) |

Bold numbers indicate statistically significant

**Table S13b: Bivariate analyses showing the association (risk ratio, 95% CI) between different neighbourhood environmental exposures and asthma-symptom trajectories ('low' vs. 'persistent high' groups) over different time points**

| Variable | Time points (age of the children) | | | | | | | |
|---|---|---|---|---|---|---|---|---|
| | 0-1 y | 2-3 y | 4-5 y | 6-7 y | 8-9 y | 10-11 y | 12-13 y | 14-15 y |
| Heavy traffic on street_ Yes | **1.35 (1.10,1.65)** | 1.20 (0.97,1.47) | **1.35 (1.10,1.67)** | **1.43 (1.16,1.75)** | **1.44 (1.17,1.78)** | **1.36 (1.09,1.69)** | 1.20 (0.95,1.51) | **1.38 (1.09,1.75)** |
| Neighbourhood liveability scale | **1.48 (1.22,1.79)** | **1.24 (1.05,1.48)** | **1.28 (1.08,1.52)** | **1.18 (1.00,1.39)** | **1.23 (1.08,1.39)** | 1.16 (0.98,1.38) | 1.10 (0.91,1.32) | 1.11 (0.93,1.34) |
| Neighbourhood facilities scale | 1.07 (0.94,1.21) | 1.00 (0.89,1.14) | **1.16 (1.02,1.31)** | 1.04 (0.92,1.18) | 1.04 (0.92,1.18) | 1.09 (0.96,1.24) | 1.06 (0.92,1.22) | 1.01 (0.87,1.17) |
| General condition of nearby buildings_ bad | 0.63 (0.25,1.06) | 1.50 (0.72,3.14) | 1.79 (0.87,3.64) | 1.56 (0.67,3.60) | 0.81 (0.34,1.92) | 1.34 (0.55,3.27) | **2.29 (1.01,5.19)** | 2.30 (0.96,5.51) |
| NDVI value of the area | 0.75 (0.34,1.63) | 0.80 (0.37,1.75) | 1.15 (0.52,2.53) | 0.83 (0.38,1.83) | 1.07 (0.48,2.38) | 0.58 (0.25,1.34) | 0.51 (0.21,1.24) | 0.67 (0.27,1.66) |

Bold numbers indicate statistically significant

Table S14: Number and percentage of participants having bad trajectories of both heavy traffic on street and neighbourhood liveability score.

| **Combination of trajectory groups** | **n (%)** |
|---|---|
| Persistently moderate heavy traffic + Moderate & static liveability | 942 (22%) |
| Persistently high heavy traffic + Moderate & static liveability | 308 (7%) |

**References (for Supplemental Texts):**


1. Jones BL, Nagin DS. A Stata plugin for estimating group-based trajectory models. *Sociological Methods & Research* 2012; **42**(4): 608-13.

2. Nagin DS. Group-based trajectory modeling: an overview. *Handbook of quantitative criminology* 2010: 53-67.

3. Sbihi H, Koehoorn M, Tamburic L, Brauer M. Asthma trajectories in a population-based birth cohort. Impacts of air pollution and greenness. *American journal of respiratory and critical care medicine* 2017; **195**(5): 607-13.

4. Nagin DS, Jones BL, Passos VL, Tremblay RE. Group-based multi-trajectory modeling. *Statistical methods in medical research* 2018; **27**(7): 2015-23.

5. Nagin DS, Odgers CL. Group-based trajectory modeling in clinical research. *Annual review of clinical psychology* 2010; **6**: 109-38.
